# Amplify Gait to Improve Locomotor Engagement in Spinal Cord Injury (AGILE SCI) trial: study protocol for an assessor blinded randomized controlled trial

**DOI:** 10.1186/s12883-024-03757-2

**Published:** 2024-08-03

**Authors:** Keith E. Gordon, Shamali Dusane, Jennifer H. Kahn, Anna Shafer, Gabrielle Brazg, Heather Henderson, Kwang-Youn A. Kim

**Affiliations:** 1 Department of Physical Therapy and Human Movement Sciences, Northwestern University, Feinberg School of Medicine, Chicago, IL 60611 USA; 2 Research Service, Edward Hines Jr. VA Hospital, Hines, IL 60141 USA; 3 Shirley Ryan AbilityLab, Chicago, IL 60611 USA; 4 Department of Preventive Medicine, Northwestern University, Feinberg School of Medicine, Chicago, IL 60611 USA

**Keywords:** Balance, Gait, Locomotor training, Walking, Error augmentation, Robotics

## Abstract

**Background:**

Among ambulatory people with incomplete spinal cord injury (iSCI), balance deficits are a primary factor limiting participation in walking activities. There is broad recognition that effective interventions are needed to enhance walking balance following iSCI. Interventions that amplify self-generated movements (e.g., error augmentation) can accelerate motor learning by intensifying sensorimotor feedback and facilitating exploration of motor control strategies. These features may be beneficial for retraining walking balance after iSCI. We have developed a cable-driven robot that creates a *movement amplification environment* during treadmill walking. The robot applies a continuous, laterally-directed, force to the pelvis that is proportional in magnitude to real-time lateral velocity. Our purpose is to investigate the effects of locomotor training in this movement amplification environment on walking balance. We hypothesize that for ambulatory people with iSCI, locomotor training in a movement amplification environment will be more effective for improving walking balance and participation in walking activities than locomotor training in a natural environment (no applied external forces).

**Methods:**

We are conducting a two-arm parallel-assignment intervention. We will enroll 36 ambulatory participants with chronic iSCI. Participants will be randomized into either a control or experimental group. Each group will receive 20 locomotor training sessions. Training will be performed in either a traditional treadmill environment (control) or in a movement amplification environment (experimental). We will assess changes using measures that span the International Classification of Functioning, Disability and Health (ICF) framework including 1) clinical outcome measures of gait, balance, and quality of life, 2) biomechanical assessments of walking balance, and 3) participation in walking activities quantified by number of steps taken per day.

**Discussion:**

Training walking balance in people with iSCI by amplifying the individual’s own movement during walking is a radical departure from current practice and may result in new strategies for addressing balance impairments. Knowledge gained from this study will expand our understanding of how people with iSCI improve walking balance and how an intervention targeting walking balance affects participation in walking activities. Successful outcomes could motivate development of clinically feasible tools to replicate the movement amplification environment within clinical settings.

**Trial registration:**

NCT04340063.

**Supplementary Information:**

The online version contains supplementary material available at 10.1186/s12883-024-03757-2.

## Introduction

Among ambulatory people with incomplete spinal cord injury (iSCI), balance deficits are common [1] and are inversely related to functional walking ability [2–6] and participation in walking activities (i.e., number of steps per day) [7]. In this population, balance is a better predictor of participation in walking activities than lower-extremity muscle strength, spasticity, balance confidence, or metabolic efficiency [7]. In addition, balance deficits may contribute to high fall rates [8]. Approximately 78% of ambulatory people with iSCI fall annually [9] with most falls occurring during walking [10]. This population tends to avoid walking in challenging environments [11], and fatigues rapidly during walking due to the use of metabolically inefficient strategies used to maintain balance [12]. Restricted participation in walking activities due to balance deficits may limit social engagement and activities of daily living [13]. The effects of balance on participation in walking activities is particularly concerning as people with iSCI average only 2,600 steps per day [7], well below the sedentary threshold of 5,000 steps [14]. Thus, effective evidence-based interventions are needed to enhance walking balance among people with iSCI [15–17].

While intensive gait training interventions have shown promise in improving overground walking ability of people with iSCI classified as C or D on the American Spinal Injury Association (ASIA) Impairment Scale (AIS) [18–20], the impact of these interventions on balance have mixed outcomes [21, 22]. For people with iSCI, deficits in walking balance pose a significant risk for falls and subsequent injury, as well as contribute to decreased participation in activity [8, 10, 23]. Surprisingly, there has been relatively little research examining methods to improve walking balance after iSCI [17, 24–26]. A recent clinical trial involving people with chronic iSCI found similar improvements in reactive balance following intensive balance training (challenging static and dynamic tasks) with and without supplementary manual external balance perturbations [15, 27]. In addition, the trial found evidence that perturbation training may help reduce fall rates. This research highlights that intensive balance training, regardless of exposure to external balance perturbations, is effective for improving reactive balance in people with iSCI. In another study, people with iSCI underwent precision training (stepping over obstacles of different heights and targets of different sizes) focusing on dynamic balance and skill during walking along with endurance training focusing on walking speed [25]. The study findings indicated improvement in overground walking suggesting effectiveness of such targeted interventions [25, 26]. Given the limited availability of robust evidence, there exists a noticeable gap in the literature regarding effective walking balance training methods. To address this knowledge gap, our current clinical trial will examine a novel gait training intervention targeting the anticipatory component of walking balance in people with iSCI.

The basis for our approach to train walking balance is that interventions that amplify self-generated movements (e.g., error augmentation or movement amplification) can enhance sensorimotor feedback [28] and facilitate motor exploration [29], which in turn can accelerate motor learning and skill acquisition [30, 31]. Specifically, our central hypothesis is that gait training performed in a movement amplification environment will be more effective for improving walking balance and participation in walking activities than locomotor training in a natural environment (no supplemental external forces applied) in people with iSCI. To explore this idea, we have created a training environment to amplify a participant’s own mediolateral whole-body center of mass (COM) motions during walking. Lateral motion is amplified using a cable-driven robot that applies smooth continuous forces to the pelvis that are proportional in magnitude and direction to the user’s real-time lateral velocity [32–35] (i.e., when a person moves to their right, the robot applies forces that accelerate the person to their right). The focus is on medio-lateral motion because the requirement of the nervous system to successfully control frontal plane balance during walking are believed to be considerable in comparison to fore-aft motion (sagittal plane), which benefits from stabilizing body segment mechanics [36–38]. In addition, the ability to control lateral COM motion during walking has been found to correlate with several clinical gait and balance measures in people with iSCI [39], suggesting functional relevance.

Our movement amplification environment is similar in principle to error augmentation methods that have been shown to enhance experience-based learning of reaching movements [30], leg swing trajectories [40], and walking symmetry [41]. The augmentation of sensorimotor feedback makes it easier for the nervous system to detect small movement errors, and to learn the mapping between a given motor command and the resulting motion [28]. Additionally, movement amplification may aid in the acquisition of new motor patterns by encouraging movement exploration and providing experience with a greater range of movement velocities than during movement in a natural environment [29]. While error augmentation and movement amplification approaches have shown promise for retraining control of reaching movements in neurologic populations [30, 31], the use of this approach to improve walking balance in people with iSCI has not been examined. Training balance during locomotion in people with iSCI by amplifying their own self-generated COM motion is a radical departure from current practice. If successful, this trial could motivate the development of new clinical tools and treatment approaches.

The purpose of this clinical trial is to investigate if locomotor training in a movement amplification environment can effectively improve walking balance and increase participation in walking activities of ambulatory people with iSCI. We are using a two-arm parallel-assignment intervention design where participants are randomized into a control group receiving a moderate to high-intensity treadmill-based locomotor training performed in a natural environment [21] or an experimental group receiving a matched intervention performed in a movement amplification environment. Evaluation of the two groups at different time points throughout the intervention include clinical outcome measures to assess changes in walking balance, quantitative biomechanical gait assessments, and daily stepping activity using activity monitors [42, 43]. We hypothesize that improvements in both walking balance and daily stepping activity will be greater in the experimental group than control group.

## Methods

### Trial design

This study is a single-site, assessor-blinded, two-arm randomized clinical trial. The Institutional Review Boards of the Edward Hines Jr. VA Hospital and Northwestern University approved the protocol on February 10, 2020, and April 23, 2020, respectively. Any protocol modifications will be approved by the local IRB committees and communicated to the study sponsor, and enrolled participants. Protocol modifications will also be updated in clinical trial registry. Data collection began on September 28, 2020 at the Human Agility Laboratory, Northwestern University, Chicago. All participants provide written informed consent prior to enrollment. An approved study team member will obtain informed written consent from all participants in the study. Baseline assessment is completed followed by stratification of participants by preferred overground walking speed and device use (walks ≥ 0.5 m/s without the use of any assistive device or < 0.5 m/s and/or requiring an assistive device), and then randomized into either an experimental or control group. Each group receives 20 moderate to high-intensity, treadmill-based locomotor training sessions. The experimental group trains in a movement amplification environment while the control group trains in a conventional treadmill environment. Re-assessments are administered following the 10th training session, within 1-week of the 20th training session, and 3-months after the 20th training session.

### Participants

**Fig. 1:**
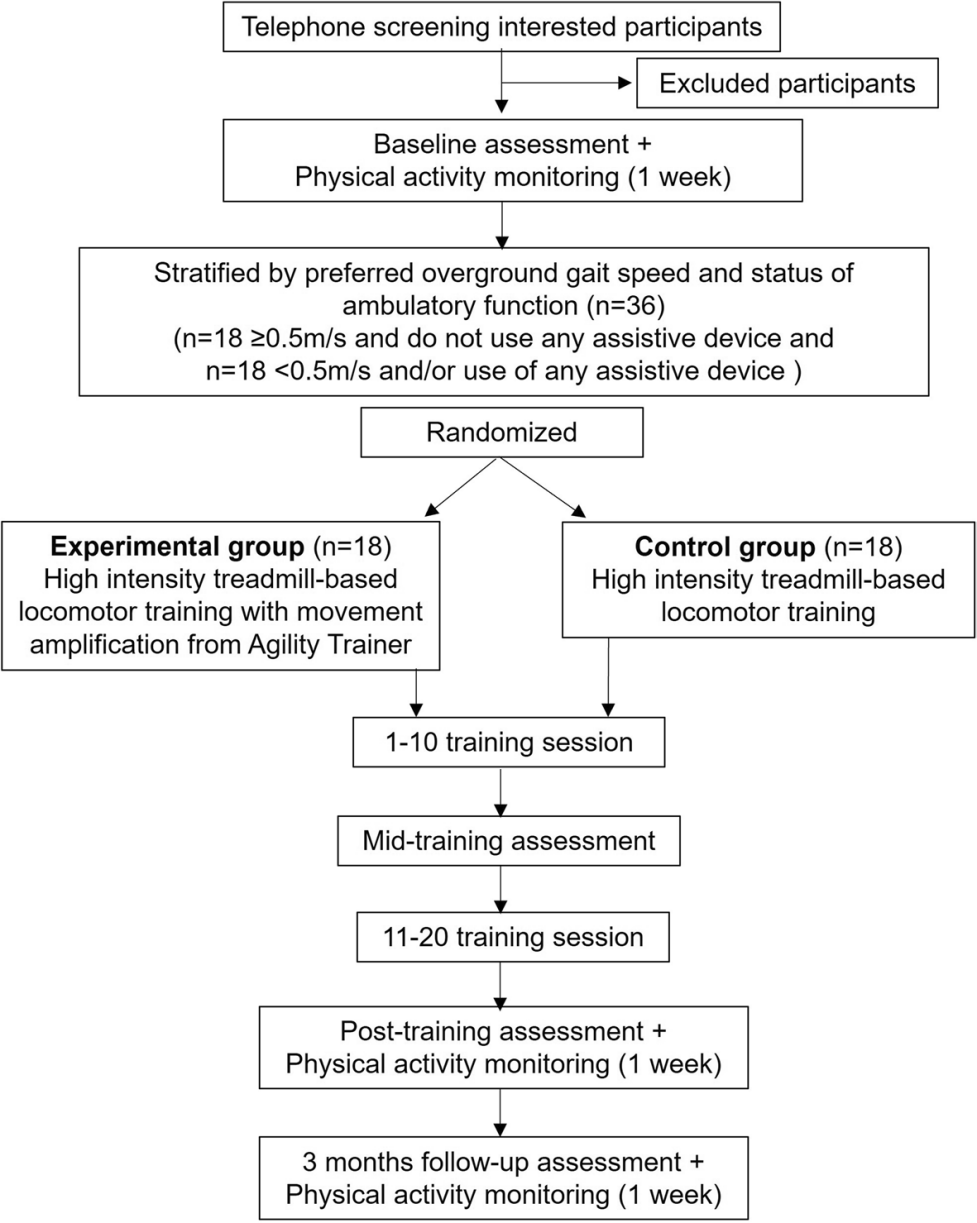
Study design flowchart. Following a telephone screening, participants undergo the consenting process and in-person screening to confirm their eligibility. Enrolled participants undergo a baseline assessment and have their physical activity monitored for one week before beginning locomotor training. Participants are stratified based on their baseline overground preferred walking speed and use of assistive devices and then randomly assigned to either the experimental or control group. Both groups undergo high intensity treadmill-based locomotor training for a total of 20 sessions. The experimental group completes locomotor training within the movement amplification environment created by the Agility Trainer. Mid-training (after training session ten), post-training (after training session 20) and follow-up (three months after training session 20) assessments are performed by a licensed physical therapist blinded to group allocation. Physical activity monitoring is performed for one week immediately after the 20th training session and 3-months following the 20th training session

Thirty-six adults with iSCI are being recruited using flyers, via clinical staff at the Edward Hines Jr. VA Hospital and the Shirley Ryan AbilityLab, presentations at SCI support groups, and through a Northwestern University/Shirley Ryan AbilityLab maintained Clinical Research Registry for SCI. The registry provides demographics and medical history of people with iSCI who have consented to be contacted to participate in research. Interested participants undergo a telephone screening and receive medical clearance from their physician prior to enrollment (Fig. 1). An in-person screening is also performed to verify that the participant meets all inclusion/exclusion criteria (Table 1).

**Table 1:** Inclusion and exclusion criteria for study participation

Inclusion criteria	Exclusion criteria
a) 18 to 80 years of age b) Medically stable with medical clearance from a physician to participate c) Neurologic level of the SCI between C1-T10 with American Spinal Injury Association (ASIA) Impairment Scale (AIS) C or D d) > 6 months since initial injury e) Passive range of motion of the legs within functional limits and not restricting the ability to engage in locomotor training f) Able to ambulate 10 m without physical assistance or while using assistive devices such as single cane or rolling walker	a) Excessive spasticity in the lower limbs as measured by a score of > 3 on the Modified Ashworth Scale b) Inability to tolerate 30 min of standing c) Severe cardiovascular and pulmonary disease d) History of recurrent fractures or known orthopedic problems in the lower extremities (i.e., heterotopic ossification) e) Concomitant central or peripheral neurological injury (i.e., traumatic head injury or peripheral nerve damage in lower limbs) f) Inability to provide informed consent due to cognitive impairments g) Presence of unhealed decubiti or other skin compromise h) Enrollment in concurrent physical therapy or research involving locomotor training i) Use of braces/orthotics crossing the knee joint j) Known pregnancy

Before enrolling any participants in the trial, a study team member, who is not involved in recruitment or assessment of walking speed/assistive device use, used a random number generator to produce two random sequences of study group assignments (experimental or control). Each sequence consisted of 20 total assignments and had a balanced number of experimental control group assignments. A specific sequence was then assigned to each stratification group (participants who walk ≥ 0.5 m/s without the use of any assistive device or < 0.5 m/s and/or requiring an assistive device). The sequences were then sealed in an envelope that was only accessible by another study team member who was not involved in recruitment or assessment of walking speed/assistive device use. Following stratification, this study team member then assigned the participant to a study group based on the random sequence.

### Experimental setup

Locomotor training is conducted on a large treadmill (2.62 m long × 1.45 m wide) (Tuff Tread, Willis, TX), providing space to perform lateral maneuvers. For safety, participants wear a trunk harness attached to a passive overhead safety support (ZeroG, Aretech, USA). Safety support straps are adjusted to allow travel across the treadmill and bodyweight support is not provided. During training, participants wear a Polar H10 heart rate (HR) monitor (Polar Electro UK Ltd., Warwick, UK) around their chest to record and display real-time HR. A large monitor positioned in front of the treadmill displays training session time, treadmill speed, real-time HR, and images that can be used to facilitate various balance-tasks. A StepWatch4 activity monitor (Modus, Edmonds, WA) is worn around the participants preferred ankle to measure the number of steps during the training session.

**Fig. 2:**
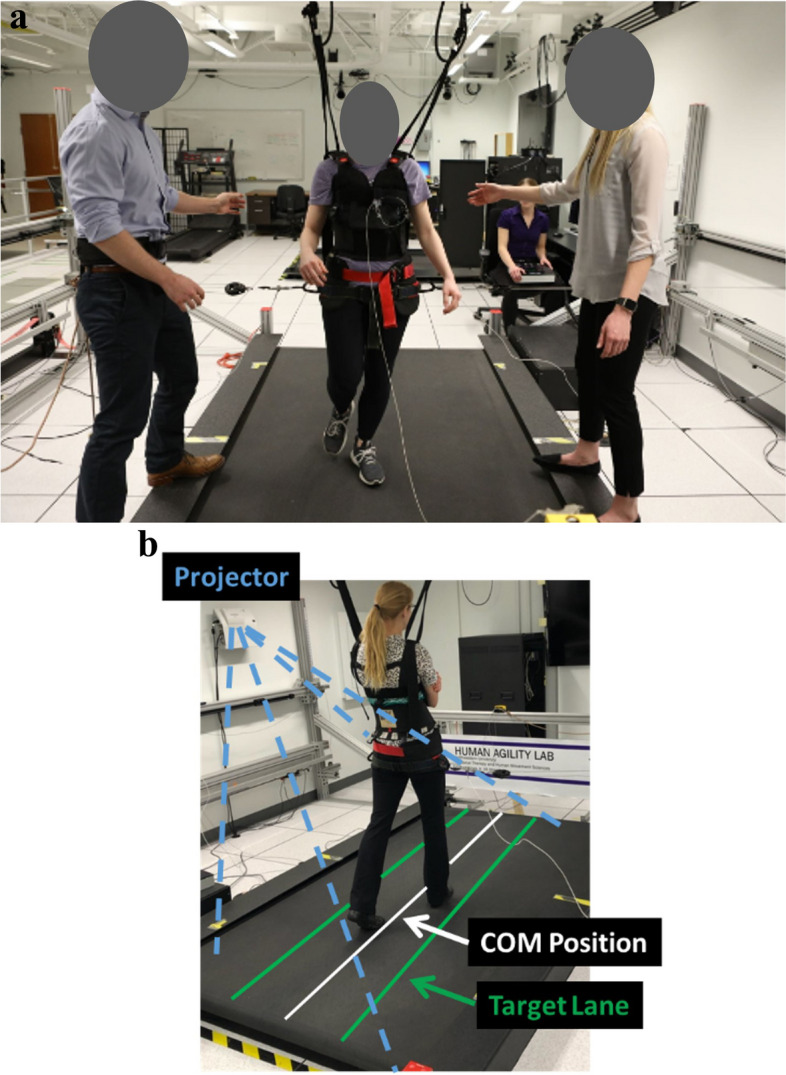
Experimental set-up. **a**) The Agility Trainer, a cable-driven robot applies lateral forces to the pelvis to create the movement amplification environment during treadmill walking. **b**) A biomechanical gait assessment is performed to determine improvement in maximum ability of participants to control the lateral motion of their center of mass (COM) position. Real-time medio-lateral COM position of the participant is projected on the length of the treadmill surface. Participants are instructed to walk at their preferred treadmill speed and to do their best to keep the white line representing their medio-lateral COM position within the green target lane. If successful, the target lane width will progressively decrease. If not, the area outside of the target lane (to either the left or right) changes to red, providing immediate visual feedback that the participant has made an error and should attempt to return their COM to the lane immediately. The target lane width will also progressively increase with continued errors

Participants in the experimental group complete locomotor training sessions in a movement amplification environment created by the Agility Trainer, a custom-built cable-driven robot. (Fig. 2a and Supplemental Video 1) [33–35]. It creates this environment by applying a net lateral force to a participant’s pelvis that is proportional in magnitude and in the same direction as the participant’s real-time lateral COM velocity [32]. A smooth continuous force is applied to the pelvis such that when a participant moves to the right or left, the applied force proportionately accelerates the participant in the same direction. The Agility Trainer consists of two series elastic actuators and a cable system routed through a series of pulleys and trolleys connected to each side of a snug pelvic harness. Load cells in series with the cables and position-sensing optical encoders within the series elastic actuator provide feedback input to the nested proportional-derivative controller that generates the amplification environment through tension in the cables [32]. The system is controlled using a cRIO-9045 FPGA with LabVIEW Real-Time software (National Instruments, Austin, TX). As a cable-driven system, it is necessary to maintain a minimum baseline tension to avoid slack in the cables. The baseline tension in the cables allows for free fore-aft motion and minimal restriction of normal pelvis motion during gait. For the movement amplification environment, the participant’s lateral velocity, *vel*, is proportionately multiplied by the field gain, *b*, to yield a negative viscous force, *F*_*v*_, vector in the same direction as the velocity, see Eq. (1).

1 $${F}_{v}=b*vel$$

Further details on how the negative viscosity force field is generated as well as verification that the system is sufficiently able to generate forces to substantially accelerate the COM of a person (up to ~ 15% bodyweight) and operate at bandwidths greater than the frequency of lateral COM movements during walking can be found in Brown et al. [32]. For this trial, the field gain of the movement amplification environment is adjusted from -25 (less challenging) to -50 (more challenging) based on participant performance. Total applied output forces in each cable are capped at 100 N for safety and to avoid overstretching of the extension spring. Additional safety features include mechanical stops to ensure the device cannot move participants beyond the lateral treadmill boundaries and multiple emergency shut-off switches to rapidly disengage the device.

### Locomotor training intervention

Both groups receive 20 sessions of moderate to high-intensity locomotor training on a treadmill. The goal is to conduct two training sessions per week on non-consecutive days. The training sessions are directed by a licensed physical therapist and consist of 45 min total time on the treadmill (including rest breaks as needed). The goal of each session is to achieve 40-min of walking practice within a target HR range and rating of perceived exertion (RPE) using the Borg RPE Scale. Every session begins and ends with a 2.5-min warmup and cool down, respectively, wherein participants complete treadmill walking at a target HR of 50 to 65% of their estimated age-predicted HR maximum (APHRmax). APHRmax is calculated as 208 – (0.7 × age) [44]. The 40-min body of the training session is split into four 10-min segments that focus on either speed (two segments) or balance (two segments). The order of the speed and balance segments alternate within a session, and the starting segment alternates between sessions. Target HR during the speed and balance segments is 70 to 85% of APHRmax. If the target HR cannot be achieved, RPE, a secondary measure of intensity, is used with a target exertion of 15 “hard” to 17 “very hard,” on the 20-point rating scale [45]. Based on the participant’s tolerance and performance, the physical therapist modifies the training tasks to achieve the target HR and/or RPE.

The objective during speed segments is to increase peak walking speed and endurance at fast speeds while maintaining the target HR and RPE. This is achieved using interval training periods of 30-s to several minutes during which the speed is varied between faster speeds that challenge the participant’s ability and slower speeds that allow for an active recovery period. The objective during the balance segments is to maximize repetitions of treadmill walking during exposure to a variety of tasks that challenge walking balance (Table 2 and Supplemental Video 1) [46]. Both speed and balance training are progressed within and across training sessions to continuously challenge participants’ abilities. At the physical therapist’s discretion, participants are provided with hand-held assistance as necessary to safely complete the desired stepping tasks with an overall goal to minimize external support. During training, participants do not have access to handrails or other forms of external assistance but are allowed to use any passive ankle–foot orthoses that they typically wear during community ambulation.

**Table 2:** Tasks used to challenge balance during gait training

Task	Task Description	Task Progression
Reducing Manual assistance	Participants using bilateral handheld assistance during walking are challenged to walk with unilateral handheld assistance that alternates between the left and right sides	Unilateral handheld assistance is gradually reduced to no external assistance
Lateral stepping maneuvers	Physical therapist cues the participant to move from one side of the treadmill to the other by crossing a line projected along the length of the treadmill. Maneuvers are performed at varying speeds during forward walking	Increase the frequency of lateral maneuvers performed
Head turns	Participants perform cued vertical and horizontal head turns while maintaining forward walking	Increase the magnitude and duration of the head turns
Obstacle negotiation	Participants are asked to step over small objects (i.e., bean bags) during forward walking	Increase the frequency of obstacles with variable placement
Altered stepping	Participants are asked to perform high knee marching or heel kick walking (maximal knee flexion during swing phase)	Modify the speed of the treadmill and minimize normal stepping between bouts
Cognitive and visual focus dual task	Images (pictures, letters, numbers, etc.) are shown on a monitor mounted in front of the treadmill to divert participants gaze away from their feet. Participants also engage in various cognitive tasks related to the projected images and/or giving mathematical addition/subtraction tasks	Increase the speed of the changing images, increase the complexity of the cognitive task being requested (i.e., name object > name the letter that the object starts with > name the last letter of the object > spell the word)
Walking with a narrow base of support	Participants are asked to walk on a straight line projected on the treadmill and continue to place foot ahead of each other	Participants are asked to perform tandem walking wherein toes of the first foot touch the heel of the next foot at each step
Backward walking	Participants are asked to walk backwards	Walking speed is increased gradually, and additional balance challenges such as lateral stepping maneuvers, or altered stepping are added based on participants’ tolerance

### Experimental group intervention

Participants in the experimental group complete the described locomotor training within a movement amplification environment created by the Agility Trainer. The movement amplification gain is gradually increased within and across sessions to increase the challenge for participants to control their lateral COM motion during walking. The initial field gain of the movement amplification environment is set as low as -25. If participants successfully control their walking in the movement amplification environment without repeated failures (loss of balance requiring physical assistance from physical therapist to recover), the field gain is incrementally increased by -5, with a maximum increase per session of -10 above a participants’ prior maximum. Field gains are increased until a maximum gain of -50 is reached. Total applied lateral force will be proportional to the participant’s velocity but is capped at 100N.

### Control group intervention

Participants in the control group receive the above-described locomotor training in a natural environment (no movement amplification) using the same treadmill, safety support and setup as the experimental group.

### Safety during assessment and training sessions

A study team member records participants’ vital signs and RPE before, during, and after each training session. The physical therapist continually monitors participants for any evidence of adverse or unanticipated events or presence of discomfort during both assessments and training sessions. To prevent falls, a safety harness and gait belt are used during treadmill walking and overground walking (assessments only), respectively.

### Documentation of training sessions

Participants’ vital signs (HR and BP) and rating of perceived exertion using the Borg RPE scale are recorded before, during, and after the training. The following is recorded during the session: walking speeds, time for which each speed was maintained, number of times physical assistance (from the physical therapist or overhead safety support) was provided to prevent a fall (falls are defined as inability to recover loss of balance without physical assistance), balance-challenging tasks completed, and movement amplification environment gains (if applicable). After each training session the total walking time, peak RPE, total number of steps (StepWatch, Modus, Washington DC) and amount of time spent in moderate (50–65%) and high (70–85% and > 85%) exercise intensity based on percentage of APHRmax are recorded [44, 47].

### Assessments

Data is collected using a combination of performance-based and self-report measures that span each level of the International Classification of Function, Disability, and Health (body structure and function, activity, and participation) [48]. Participants’ demographic information and a brief medical history is collected which includes demographic variables (age, sex, date of birth), date of SCI, level of SCI, cause of SCI, current and past medical history, current medications, current ambulatory ability in the home and community, use of any assistive devices during ambulation and self-reported number of falls in the past year. Body structure and function, and activity are assessed using clinical outcome measures and biomechanical gait assessments, while participation in activity is assessed using a StepWatch4 activity monitor. The physical therapist performing the clinical assessment is blinded to the intervention group assignment.

### Clinical outcome measures

Clinical outcomes measures are assessed at four time points; baseline, mid-training, post-training, and 3 months post-training (BSL, Mid, Post, and Follow Up) (Table 3). First, the participants AIS level is confirmed with the Lower Extremity Motor Score (LEMS) assessment, a portion of the International Standards for Neurological Classification of Spinal Cord Injury (ISNCSCI) [49]. The LEMS assesses the strength of five muscle groups representing neurological levels L2 to S1. If the LEMS could not confirm the AIS, additional sensory testing for light touch and pin prick is completed to determine the level of neurological injury. Manual muscle testing of hip abductor strength is also performed, an important muscle group for controlling lateral motion.

**Table 3:** Clinical outcomes measures

Clinical outcomes	Description
Lower Extremity Motor Score (LEMS)	A portion of the International Standards for Neurological Classification of Spinal Cord Injury (ISNCSCI) [49] is used to help classify the type of SCI. The LEMS assesses strength of five muscle groups representing neurological levels L2 to S1
Hip abductor and adductor muscle strength	Manual muscle tests are performed to assess strength of hip abductors and adductors, which are important for controlling lateral motions
Walking Index for Spinal Cord Injury II (WISCI II)	Evaluates the amount of physical assistance needed for gait after SCI. It has excellent reliability and validity in the chronic iSCI population [50] and has a strong correlation with measures of balance [6]
Functional Gait Assessment (FGA)	Ten-item test that evaluates dynamic balance and postural stability during gait [51] and has been found to be valid and reliable for people with iSCI [52]
10 Meter Walk Test (10MWT)	Simple measurement of an individual's average walking speed is performed. Research indicates 10MWT as a predictor of community ambulation and a clinically useful test to determine gait changes [53, 54]. Gait speed is measured at self-selected speed (instruction: “walk at your normal comfortable pace”) and fastest-possible speed (instruction: “as fast as you safely can”)
Timed Up and Go test (TUG)	Assesses functional mobility, walking balance [55] and fall risk [56]. It is a quick, valid, reliable and widely used clinical performance-based measure [57]
Activities-Specific Balance Confidence (ABC) scale	Self-reported measure of an individual’s confidence while performing specific postural and ambulatory activities. The ABC is a reliable and valid measure of balance confidence in people with iSCI who ambulate in the community [58]
Balance Evaluations Systems Test (BESTest)	Assesses balance impairments across six different domains of postural control. Specifically, the reactive balance item from the BESTest is used to assess changes in the capacity to react to fore-aft, and lateral perturbations [59]
Berg Balance Scale (BBS)	14-item measure that assesses in place balance [4] with excellent validity and reliability in people with AIS D and ceiling effect in higher functioning individuals
World Health Organization Quality of Life scale (WHOQOL-BREF)	26-item self-report quality of life assessment focusing on areas such as physical, psychological, social and environmental health is measured [60]
International Consultation on Incontinence Questionnaire-Urinary Incontinence Short Form (ICIQ- UI SF)	4-item self-report of urinary incontinence to document changes in bladder function is used [61]

We use several clinical outcome measures to identify functional changes in gait-related balance (Table 3). The Walking Index for Spinal Cord Injury II (WISCI II**)** evaluates the amount of physical assistance needed for gait after spinal cord injury. The WISCI II has excellent reliability and validity in the chronic iSCI population [50]. The WISCI II has also been found to have a strong correlation with measures of balance [6]. The Functional Gait Assessment (FGA) is a ten-item test that evaluates dynamic balance and postural stability during gait [51]. The FGA is both valid and reliable for assessing walking balance of people with iSCI [52]. The 10 Meter Walk Test (10MWT) is a measurement of average walking speed. Research indicates that the 10MWT is a valid and reliable measure of ambulatory ability for people with SCI, has good clinical utility, and is known to be correlated with the WISCI II [53, 62]. Gait speed is measured at self-selected (instruction: “walk at your normal comfortable pace”) and fastest-possible speeds (instruction: “as fast as you safely can”). The Activities-Specific Balance Confidence (ABC) scale is a self-report measurement of an individual’s confidence that they will not lose their balance while performing numerous daily activities. The ABC is a reliable and valid measure of balance confidence in people with iSCI who ambulate in the community [58]. The Balance Evaluations Systems Test (BESTest) is used to assess balance impairments across six different domains of postural control. We will specifically use the reactive balance item from the BESTest to assess changes in the capacity to react to fore-aft and lateral perturbations [59]. The Berg Balance Scale (BBS) is a 14-item measure that assesses static balance [4]. The test has excellent validity and reliability data for people with AIS D SCI, however in higher functioning people, there is a ceiling effect. As the test does not assess dynamic walking balance it is recommended to be used in conjunction with additional measures, such as 10MWT [63]. The Timed Up and Go test (TUG) is a mobility test that measures time taken by a participant to stand up, walk ten feet, turn around, walk back, and sit down again [55]. The TUG is a commonly used screening tool to identify patients at risk of falling [56]. The TUG has been validated and found to be reliable in people with SCI [57].

Data is collected using an abbreviated version of The World Health Organization Quality of Life scale (WHOQOL-BREF) to determine the impact of our intervention on participants quality of life [60]. The WHOQOL-BREF is a 26-item self-report quality of life assessment with four domains: physical, psychological, social, and environmental health. The International Consultation on Incontinence Questionnaire-Urinary Incontinence Short Form (ICIQ- UI SF), a 4-item self-report of urinary incontinence to document changes in bladder function is used [61]. This outcome is included since two of the three participants in our case series investigating the effect of locomotor training performed in a movement amplification environment reported substantial improvements in bowel/bladder and sexual function following training. This is consistent with a previous locomotor training study that also found improvements in bladder function [64]. These improvements may be attributable to neuroplasticity or biomechanical demands related to pelvic floor and core musculature involvement in dynamic balance during locomotion.

### Biomechanical gait assessments

Biomechanical gait assessments are also conducted at four time points (BSL, Mid, Post, and Follow Up) to evaluate preferred walking mechanics and quantify the ability of participants to control their whole-body COM motion during treadmill walking. Specifically, we collect kinematic data as participants walk on an oversized treadmill, walking surface 2.6 × 1.4 m (Tuff Tread, Willis, TX). We record 3D coordinates of 19 reflective markers placed on the pelvis and lower limbs using a 12-camera motion capture system (Qualisys, Gothenburg Sweden). Participants are not allowed to use any assistive devices (canes, walkers, handrails) except passive ankle–foot orthoses that they typically wear during community ambulation. A physical therapist provides hand-held assistance to participants only as necessary to allow continuous stepping. During the BSL biomechanical gait assessment, all walking is performed at the participant’s preferred treadmill speed which is identified at the beginning of each biomechanical gait assessment session through a staircase method of increasing and decreasing the treadmill speed until desired speed is confirmed through verbal feedback. For the Mid, Post, and Follow Up assessments, walking is completed at both the current preferred treadmill speed (if preferred treadmill speed has changed with training since BSL) and at the preferred treadmill speed identified during BSL. Before beginning any kinematic assessments, participants are given two minutes to acclimate to each walking speed that will be used during the assessment.

To evaluate participants’ preferred walking mechanics, we collect kinematic data as participants perform two, two-minute walking trials at both their current preferred walking speed and their preferred walking speed identified during BSL. The order of walking speeds is randomized. During these trials, participants are instructed to walk as they feel most comfortable. No additional external support or feedback is provided during these trials.

Participants who can complete the above treadmill walking task with no manual assistance then perform a novel walking assessment, which was designed and developed to quantify participants’ maximum ability to control their lateral COM excursion during walking (Fig. 2b) [39]. During this assessment, participants receive information regarding their real time medio-lateral COM position (Hitachi, Tokyo, Japan), represented by a white line projected along the length of the treadmill surface. Lateral COM position is estimated from real-time 3D locations of the reflective markers placed on the pelvis using a 12-camera motion capture system (Qualisys, Gothenburg Sweden) and streamed to a custom-programmed control algorithm (LabVIEW, National Instruments, Austin, TX). The control algorithm calculates medio-lateral COM position [65] and transforms the data into the treadmill coordinate system for display. Additionally, lateral boundary targets for COM position (“target lane”) are projected on the treadmill (Fig. 2b). The control algorithm adjusts the width of the target lane to progressively challenge participants’ ability to control their lateral COM position (Fig. 2b). Participants are instructed to do their best to maintain their medio-lateral COM position within the target lane during treadmill walking. During walking, the area outside of the target lane changes to red if the COM moves outside the target lane, providing immediate visual feedback to return to the target lane. Again, participants’ ability to control their lateral COM position during forward walking is evaluated at the current preferred walking speed and at their BSL preferred walking speed. During the evaluation, participants do not receive any external assistance.

At each walking speed, participants are evaluated during a 63-m walking trial with rest breaks every 21 m. The starting lane width is set to 200 mm. During each trial the control algorithm dynamically adjusts the target lane width. If the participant maintains their COM position in the lane for 1.5 consecutive meters of forward walking, the lane decreases in width by 10 mm. If the participant walks for 3 m without maintaining COM position in the lane for 1.5 consecutive meters, the lane width increases by 10 mm. The minimum lane width is set at 5 mm. The algorithm thresholds were established in pilot testing prior to the current study to minimize walking effort yet converge on the smallest lane width participants could maintain. The order of walking speeds is randomized. The primary outcome of this evaluation is the minimum lateral COM excursion that the participant achieves within a 1.5 m window, evaluated every 0.5 m throughout each 21 m test. A reduction in the minimum lateral COM excursion following training would suggest improved balance during gait.

Data from all walking trials collected during assessment sessions are used to quantify COM motion and stepping characteristics. Kinematic marker data is processed using Visual3D (C-Motion, Germantown, MD) and a custom MATLAB (Mathworks, Natick, MA) program. Marker data is gap-filled and low-pass filtered (Butterworth, 6 Hz cut-off frequency). Time of initial foot contact (IC) and toe-off (TO) events are identified for each step based on fore-aft positions of the calcaneus and 2nd metatarsal markers. Medio-lateral COM position is calculated in Visual3D as the center of the Visual3D model’s pelvis. To characterize medio-lateral control of the COM during all walking trials, peak lateral COM speed and lateral COM excursion for each stride will be identified. To assess how control was instituted, we will assess mean and variability of step width, step length, step time, and minimum lateral margin of stability (MOS) of each step [66].

### Daily stepping

The average number of steps participants take per day is assessed during three separate one-week periods that occur at BSL, Post, and Follow Up using a StepWatch4 (Modus, Edmonds, WA) activity monitor worn on the ankle. This device is accurate and reliable for measuring stepping activity of people with iSCI and has been successfully used to assess daily stepping in this population [7, 42, 43]. During each assessment period, participants are asked to wear the activity monitor during all waking hours (except when bathing) for seven continuous days. Data is analyzed only for the days when the StepWatch is worn for at least 90% of waking hours. Prior research suggests that stable measures of walking activity in adults with iSCI can be obtained by averaging step count values from any two-day period in a week [43]. If participants do not wear the device for at least two full days, they are given the device for an additional seven-day period. Our measure of daily stepping is the total number of steps taken per day averaged across the number of compliant days (minimum of two) during the seven-day period.

## Statistical analysis and sample size

Our primary clinical outcome measures are the FGA, to assess functional walking balance, and the 10MWT, to assess walking speed. Our primary biomechanical gait assessment measure is the minimum lateral COM excursion during walking. Our primary measure of walking participation is the average number of steps taken per day.

To test between group differences in walking balance following intervention, we will compare changes in participants’ COM lateral excursion, calculated across the four assessment periods (BSL, Mid, Post, Follow Up) and between intervention groups (control and experimental). To account for the correlation that arises from measuring multiple data points within each participant over time (i.e., data measured from the mid- assessment period from a specific participant will bear more resemblance to data measured from the BSL- assessment period from the same participant than a different participant), we will use a linear mixed effects model. An indicator variable will be used for group assignment, 1 = experimental, 0 = control, to test for the effect of intervention while controlling for potential confounders such as initial walking speed, location of the iSCI, and age. The strength of the linear mixed effects models is that it can accommodate multiple data points from a single participant and can also manage missing data when data is missing at random. A similar strategy will be applied to examine changes in preferred walking biomechanical characteristics (average and variability of step width, step length, and minimum lateral MOS) and clinical outcome measures (10MWT, FGA, ABC etc.) across the four assessment periods and between intervention groups.

To test if changes in steps per day are different following the two interventions, changes in participants’ average steps per day across the three assessment periods (BSL, Post, Follow Up) and between intervention groups will be compared using linear mixed effects model. Potential confounders will be accounted for in the model to ensure the robustness of the findings.

We estimated our target sample size using our primary biomechanical gait assessment measure, walking balance, which we quantified by evaluating participants’ maximum ability to control mediolateral COM excursion during treadmill walking with visual feedback (Fig. 2b). We performed a power analysis to determine the minimum sample size needed to detect a 20% difference in improvements in walking balance between groups. We estimated the population variability from 13 people with iSCI performing unassisted, preferred-speed, treadmill walking who had a lateral COM excursion per stride of 0.083 ± 0.014 m (mean ± standard deviation) [67]. To estimate effect size, we used pre- and post-intervention data from pilot testing of three people with iSCI who received 16–18 locomotor training sessions performed in the movement amplification environment. These people decreased their lateral COM excursion during walking by 32–36%. Thus, at α = 0.05 and a power of 80%, we estimate that we will need a minimum sample size of 7 people per group. A recent locomotor training intervention in people with iSCI had a 12% drop out rate [21]. Thus, we plan to enroll 36 people with a conservative estimate that we will lose 20% of participants to drop out, which would ultimately yield 14 people per group.

## Adverse events

Any adverse events are reported to the Institutional Review Boards. If required, the participant can receive immediate medical care at the emergency room of either Northwestern Memorial Hospital or Edward Hines Jr. VA Hospital. The physical therapist present during the session will continually monitor the cardiopulmonary status of the participant and attend to any immediate health-related issues.

## Data safety monitoring and regulatory audits

An internal data monitoring committee consisting of the principal investigator and clinical members of the study, who were not blinded were responsible for monitoring participant safety. An external data monitoring committee was not formed for this trial. The justification for this decision was based on the relative simplicity and limited size of the proposed project. Specifically, the following factors were considered—the proposed study is low risk, members of the internal data monitoring committee are not blinded to the study interventions, the study involves less than 40 total participants, and experimental testing and training sessions are being conducted at a single location. The research compliance officer from the Edward Hines VA Jr. Hospital will conduct annual audits of the trial.

## Confidentiality

To ensure data confidentiality, all participant records will be identified with an alphanumeric code unrelated to participant name, initials, or other identifying numbers. The code will be used to prevent direct or indirect identification of study participants when reporting findings of this study. Data files will only be identified using this code number. All digital data will be kept on password-protected encrypted computers and servers. Hard copy data will be kept in a locked file cabinet in locked office space. Only the research team will have access to the digital and hard copy data. All data collected will be expressly for research and educational purposes and will not be shared with outside entities except with the explicit written consent of the participant whose data is in question. Data will be stored for 10 years after completion of the study. Only the principal investigator and sub-investigators will have access to the data during this time. The study team members who collect hard copy data during the experiment will be responsible for receipt and transmission of the data from the laboratory to a locked filing cabinet. Digital data will be backed up on an encrypted, password-protected server.

## Discussion

This trial has been designed to evaluate the effects of locomotor training in a movement amplification environment on walking balance in ambulatory people with iSCI. The movement amplification environment used in this trial was designed specifically to exaggerate the mediolateral motion of a person’s COM during treadmill walking. There are several reasons why practicing walking in a movement amplification environment may help people with iSCI to improve their walking balance more than gait training performed in a natural environment (no external forces applied). We believe there are three principal advantages of the movement amplification environment. First, the movement amplification environment will create an additional balance challenge during walking by making it more difficult to control side-to-side movements. Consistent with the concept of task specific practice (to improve a behavior, practice that specific behavior) [46], the additional challenge required to control lateral motion in the movement amplification environment may create a practice environment that directly aids in learning this important skill required for walking balance. Second, movement amplification environment, by exaggerating a person’s own movements, may enhance sensory feedback [28], providing the nervous system with information that is important for learning how a given action (e.g., providing a force against the ground) affect the resulting action (e.g., lateral motion of the COM). Finally, the movement amplification environment may increase the dynamic workspace (body positions and velocities) a person with iSCI experiences when moving [29]. To learn new motor behaviors, it is necessary to practice new movement patterns. The exaggerated movement patterns that occur when walking in a movement amplification environment may facilitate movement patterns across a larger range of body positions and velocities than a person might otherwise “explore” when walking in their natural environment. Thus, the movement amplification environment may provide a stimulus for trying new movement patterns.

We hypothesize that for ambulatory people with iSCI, locomotor training in a movement amplification environment will be more effective for improving walking balance and participation in walking activities than locomotor training in a natural environment. We will use several measures to assess the effects of a 20-session treadmill based moderate-to-high intensity locomotor training intervention performed in each environment. The assessments we will perform will allow us to quantify the independent effects of the movement amplification environment on functional measures of walking (gait speed and balance), changes in walking patterns (biomechanical assessments) during preferred walking and walking under conditions challenging control of lateral motion, and changes in the amount of walking outside of the laboratory stepping (steps per day). In addition to understanding the effects of the movement amplification environment, these assessments will provide new information that has value for understanding the effects of moderate-to-high intensity gait training on walking function in people with iSCI.

While current clinical practice guidelines recommend moderate-to-high intensity gait training to improve walking speed and distance in people with iSCI [68], the support for this recommendation comes primarily from research conducted on people with stroke. In the current trial, all participants will engage in 20-session of a moderate-to-high intensity locomotor training intervention. Outcomes from this trial will be valuable for providing support for or against the use of moderate-to-high intensity locomotor training in people with iSCI. However, it is important to acknowledge that the current trial will not include a group that receives locomotor training at lower intensities, which would be optimal for assessing the effects of training intensity on improvements in walking function (speed and balance).

Finally, while there have been many trials that clearly indicate that people with iSCI will improve their locomotor capacity (improvements in walking function including speed and independence measured in a laboratory or clinical setting) with locomotor training [19, 69–71], it is not clear how these improvements translate to increases in walking performance (how much walking a person engages in walking outside of the laboratory or clinical setting). This is a very important aspect of gait training that has not been fully considered for people with iSCI. There is an assumption that improvements in walking capacity should result in associated increases in walking performance. However, outcomes in other non-iSCI populations (e.g., stroke) suggest that the improving walking capacity may not result in proportional increases in walking performance [72] as there are several other variables that may influence changes in a person’s behavior. For people with iSCI, it is also possible that improvements in walking capacity may not directly translate to increases in how much a person walks outside the laboratory. In the current trial we will assess changes in how many steps people with iSCI take before and after training. This novel information will be valuable for understanding the relationship between gait training, walking capacity, and walking performance in people with iSCI.

This trial does have several limitations. The Agility Trainer used to create the movement amplification environment is a custom-built device that is not commercially available. If the movement amplification environment is successful, translation to the clinic would require further commercial development. Also, the Agility Trainer can only be used on a treadmill, this will limit the types of gait training activities that are used in this trial to those that can be conducted on a treadmill. The number of daily steps during home or community activity, will be monitored at discrete time points that might be influenced by factors such as weather or COVID precautions, that are difficult to control for and could influence the amount of walking participants engage in during the assessment periods.

This randomized controlled trial will examine a novel and promising method to enhance dynamic balance of people with iSCI. Positive outcomes will support development of clinically feasible tools to replicate the movement amplification environment within clinical settings. The knowledge gained from this study will expand our understanding of how targeted dynamic balance training impacts participation in walking activities in people with iSCI.

### Supplementary Information

Supplementary Material 1. Supplemental Video 1. The video shows the experimental setup used for high intensity locomotor training in a movement amplification environment for our study participants. The cables of the Agility Trainer are attached on both sides of the pelvic harness donned by a study team member with no known neuromuscular impairments (for demonstration purposes) followed by walking under the supervision of a licensed physical therapist. The session begins with a speed training block consisting of challenging fast walking followed by recovery at a slower speed. The subsequent balance training block shows the team member performing lateral maneuvers across a center line projected on the treadmill belt. This is followed by obstacle negotiation wherein the team member is instructed to walk towards the obstacle presented at different positions on the treadmill and step over it. The team member then performs targeted maneuvers (sideways, forward, backward, diagonal) moving quickly to boxes projected on the treadmill as cued by physical therapist. During tandem walking, the team member is instructed to place both feet on the projected central line (heel to toe walking). During backwards walking, initially the team member walks at a slower speed followed by walking at a faster speed and concurrently performing lateral maneuvers. Further progression of backward walking includes catching bean bags while performing a tandem walking task. Lastly the display monitor shows the participant’s heart rate which provides the physical therapist with real-time feedback on the participants cardiovascular intensity during training. It also shows the time and the training block that the participant is in. The display monitor is also used for simple cognitive tasks such as recognizing images on the screen, enlisting words starting with the letter displayed and simple math tasks. The Borg scale (6-20) on the wall adjacent to the display monitor provides the participant a method to rate their perceived exertion throughout the training session.

Supplementary Material 2.

## Data Availability

No datasets were generated or analysed during the current study.

## Abbreviations

iSCI: Incomplete spinal cord injury
AIS: American Spinal Injury Association (ASIA) Impairment Scale
COM: Center of mass
HR: Heart rate
APHRmax: Age-predicted HR maximum
RPE: Rate of perceived exertion
LEMS: Lower Extremity Motor Score
ISNCSCI: International Standards for Neurological Classification of Spinal Cord Injury
WISCI II: Walking Index for Spinal Cord Injury II
FGA: Functional Gait Assessment
10MWT: 10 Meter Walk Test
ABC scale: Activities-Specific Balance Confidence scale
BESTest: Balance Evaluations Systems Test
BBS: Berg Balance Scale
TUG: Timed Up and Go test
WHOQOL-BREF: World Health Organization Quality of Life scale
ICIQ-UI SF: International Consultation on Incontinence Questionnaire-Urinary Incontinence Short Form
MOS: Minimum lateral margin of stability
BSL: Baseline assessment
Mid: Mid-training assessment after the 10th training session
Post: Post-training assessment after the 20th training session
Follow Up: Assessment at 3-months after the 20th training session
